# Delayed treatment of cynomolgus macaques with a FVM04/CA45 monoclonal antibody cocktail provides complete protection against lethal Sudan virus infection

**DOI:** 10.1128/jvi.01242-23

**Published:** 2024-07-16

**Authors:** Mable Chan, Bryce M. Warner, Jonathan Audet, Douglas Barker, Nikesh Tailor, Robert Vendramelli, Thang Truong, Kevin Tierney, Amrit S. Boese, Honguy Qiu, Frederick W. Holtsberg, Javad Aman, Shantha Kodihalli, Darwyn Kobasa

**Affiliations:** 1 Special Pathogens program, National Microbiology Laboratory, Public Health Agency of Canada, Winnipeg, Canada; 2 Research and Development, Emergent BioSolutions Canada, Winnipeg, Manitoba, Canada; 3 Integrated BioTherapeutics, Rockville, Maryland, USA; 4 Department of Medical Microbiology and Infectious Diseases, University of Manitoba, Winnipeg, Canada; Universite Laval, Quebec, Canada

**Keywords:** filovirus, Sudan virus, monoclonal antibody, immunotherapy, nonhuman primate

## Abstract

Sudan ebolavirus (SUDV) is a member of the genus *Ebolavirus* (Family *Filoviridae*) and has caused sporadic outbreaks of Ebola disease (EBOD), or more specifically Sudan virus disease (SVD), with high mortality rates in Africa. Current vaccines and therapies that have been developed for filoviruses are almost all specific for Ebola virus (EBOV; of the species *Zaire ebolavirus*), and there is a current lack of therapeutics specific for SUDV. The recent SUDV outbreak in Uganda, which was distributed across multiple districts, including Kampala, a densely populated urban center, highlights the critical need for the development of novel SUDV-specific or pan-Ebola virus therapeutics. Previous work has characterized two monoclonal antibodies, FVM04 and CA45, which have neutralization capabilities against both EBOV and SUDV and have shown protective efficacy in animal challenge studies. Here, we expand upon this work, showing that treatment with a monoclonal antibody cocktail consisting of FVM04 and CA45 provides full protection against lethal SUDV infection in cynomolgus macaques. Studies that evaluate outcomes at late time points after infection, once clinical signs of illness are apparent, are vital for assessing the therapeutic efficacy of antibody therapeutics. We have shown that when treatment is initiated as late as 5 days after infection, with a second dose given on day 8, that treated groups showed few clinical signs or morbidity, with complete survival. This work provides further evidence that FVM04 and CA45 have strong therapeutic potential against SUDV and their development as a pan-Ebola virus therapeutic should be pursued.

**IMPORTANCE:**

There are currently no approved vaccines or therapeutics for Sudan virus, a filovirus which is highly related to Ebola virus and causes similar disease and outbreaks. In this study, a cocktail of two potent monoclonal antibodies that effectively neutralize Sudan virus was tested in a nonhuman primate model of Sudan virus disease. Treatment was highly effective, even when initiated as late as 5 days after infection, when clinical signs of infection were already evident. All treated animals showed complete recovery from infection, with little evidence of disease, while all animals that received a control treatment succumbed to infection within 8 days. The study further demonstrated the strong therapeutic potential of the antibody treatment and supported further development for use in Sudan virus outbreaks.

## INTRODUCTION

Sudan virus (SUDV) (Family *Filoviridae,* Genus *Ebolavirus*) was discovered in 1976 following an outbreak of hemorrhagic fever disease in Sudan (now South Sudan) (1). Around the same time, an outbreak of Ebola virus (EBOV) was underway in Zaire (now the Democratic Republic of the Congo) (2). These two initial outbreaks resulted in case fatality rates of 53% and 88% for SUDV and EBOV, respectively (1, 2). Since both viruses emerged, there have been outbreaks throughout sub-Saharan Africa with high case fatality rates, driven mostly by EBOV. Four other members of the *Ebolavirus* genus have been discovered and characterized, with varying capacities to cause disease in humans. Bundibugyo virus, Tai Forest virus, Reston virus, and Bombali virus have been described; however, of these four, only Bundibugyo and Tai Forest viruses have caused known disease in humans (3). The largest filovirus outbreak to date was the West African EBOV outbreak of 2013–16, which saw more than 28,000 cases and 11,000 deaths, mainly in Liberia, Guinea, and Sierra Leone (4, 5). While SUDV has previously caused fewer, more sporadic outbreaks compared with EBOV, these outbreaks saw high case fatality rates. The recent Sudan virus disease (SVD) outbreak in Uganda was declared by local authorities on 20 September 2022 and ended on 12 January 2023 causing 55 deaths with an estimated CFR of 38.7% (6).

Though there has been ongoing work toward vaccines and therapeutics for EBOV for more than 20 years, the 2013–16 West African outbreak resulted in a heightened awareness of the need for medical countermeasures against EBOV and other ebolaviruses. Prior to the West African outbreak, vaccines and therapeutic monoclonal antibodies had been developed against EBOV and shown preclinical evidence of efficacy (7, 8) and safety in phase 1 clinical trials (9). Both the vesicular stomatitis virus (VSV)-based EBOV vaccine and ZMAPP, a therapeutic cocktail of monoclonal antibodies specific for EBOV, underwent clinical studies during the outbreak (10, 11). VSV-EBOV, now licensed and named Ervebo, was shown to have high effectiveness against Ebola virus disease (EVD) (10). Treatment of Ebola virus infections with ZMAPP showed modest efficacy but demonstrated the potential and practicality of the application of antibody therapeutics for outbreak management (11). Since that time, the development of additional monoclonal antibodies with reactivity against EBOV, ebolaviruses, and filoviruses has been an intense area of research resulting in the recent testing of additional antibody therapeutics and approval of antibody cocktails for Ebola virus, including Inmazeb (REGN-EB3; atoltivimab/maftivimab/odesivimab developed by, and a registered trademark of, Regeneron Pharmaceuticals Inc.) and Ebanga (ansuvimab-zykl; formerly mAb114, developed by the Vaccine research center and US National Institutes of Health and produced by, and is a trademark of, Ridgeback Therapeutics L.P.) (12–14). Ideally, candidate therapeutics would include antibodies that can recognize conserved regions of the viral glycoprotein (GP) across different ebolaviruses or even all filoviruses.

Previous work has identified two chimeric monoclonal antibodies derived in non-human primates (NHPs), containing human Fc domains, that are capable of binding distinct sites which are conserved between EBOV and SUDV glycoproteins (GP). FVM04 targets the receptor binding site of ebolavirus GPs and can prevent the interaction of GP with the receptor Neimann-Pick C1 (NPC-1), while CA45 binds to an internal fusion loop of the GP that is conserved across ebolaviruses (15, 16). Detailed characterization of each of these antibodies has been reported, with both showing neutralizing capability against EBOV, SUDV, and Bundibugyo virus highlighting their potential as pan-ebolavirus therapeutics (15, 17). FVM04 treatment alone following EBOV or SUDV infection can provide 100% protection from lethal disease in mice, depending on the timing of administration and the dose of antibody (15, 18). In guinea pigs, FVM04 treatment alone showed reduced efficacy against EBOV but was fully protective against SUDV (15, 18). Similar data have been reported for treatment with CA45 alone, with a reduction in lethal outcomes in EBOV and SUDV-infected mice. CA45 monotherapy also provided partial protection in guinea pigs infected with guinea pig-adapted (GPA)-EBOV, but full protection against GPA-SUDV (17). Combined FVM04 and CA45 treatment was more effective in guinea pigs, and a mouse model of SUDV infection, resulting in 100% survival in post-infection treatment studies (17, 19). Treatment with a cocktail of FVM04 and CA45 also provided full protection from lethal disease in Bundibugyo virus-infected ferrets (17). In rhesus macaques, FVM04 and CA45 cocktail treatment beginning on day 4 post-infection provided 100% protection against both EBOV and SUDV. This provided evidence of the utility of this combination as a post-exposure treatment option for EBOV and SUDV infections; however, while EBOV infection was uniformly lethal in control animals in that study, only 50% of SUDV-infected control animals succumbed to disease (19).

In the current study, we examined the protective efficacy of FVM04 and CA45 cocktail treatment in SUDV-infected cynomolgus macaques, a stringent, fully lethal model of infection. Groups of infected macaques received two doses of antibody cocktail, 3 days apart, beginning on either day 4 or 5. Both regimens resulted in complete protection from lethal SVD, while PBS-treatment animals all succumbed to infection by day 10. Treatment with FVM04/CA45 in addition to preventing lethal disease also significantly reduced morbidity in SUDV-infected animals, even with delayed time to treatment. This expanded treatment window highlights the advantage of this treatment approach, as it can be given even after the virus has had significant time to replicate. Our results provide strong support for the development of FVM04 and CA45 as a broad-spectrum therapeutic approach for SVD.

## RESULTS

### Neutralization of SUDV by FVM04 and CA45

**Fig 1:**
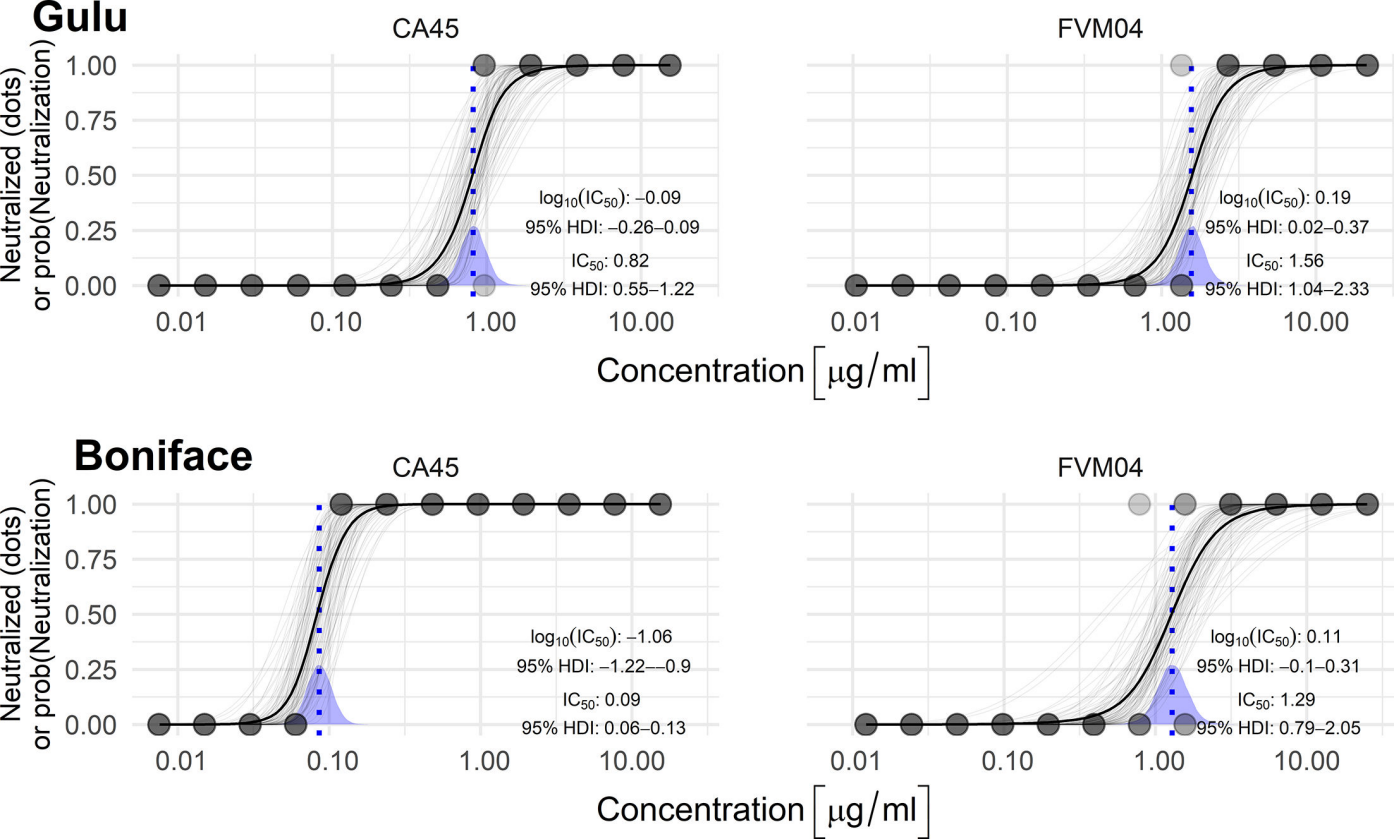
Neutralization of SUDV by FVM04 and CA45 monoclonal antibodies. *In vitro* neutralization curves showing the neutralizing ability of either FVM04 and CA45 against SUDV-Boniface or SUDV-Gulu. The thick line represents the best-fit line while the thin lines are drawn from the posterior distribution and represent the uncertainty of the fit. The dots along the top and bottom represent the proportion of the four wells that had neutralization (top) or no neutralization (bottom). The vertical dashed line represents the best estimate of the median inhibitory concentration (IC50). HDI, highest density interval.

We confirmed the *in vitro* neutralization capabilities of both FVM04 and CA45 monoclonal antibodies against SUDV strain Boniface and Gulu in a microneutralization assay. For FVM04, neutralization was similar for both strains, with calculated IC_50_ values of 1.56 [95% highest density interval (HDI): 1.04–2.35] and 1.29 (95% HDI: 0.81–2.09) µg/mL for Gulu and Boniface, respectively (Fig. 1). Interestingly for CA45, there was nearly a 10-fold difference in neutralization between the two viruses, with an IC_50_ against SUDV-Gulu of 0.82 (95% HDI: 0.55–1.23) µg/mL and an IC_50_ of 0.09 (95% HDI: 0.06–0.13) µg/mL against SUDV-Boniface (Fig. 1). CA45 showed a 2-fold and 14-fold higher neutralization activity against SUDV-Gulu and SUDV-Boniface, respectively, compared to FVM04 indicating CA45 is the more potent neutralizer *in vitro*.

### Monoclonal antibody cocktail treatment protects against severe disease

Due to their potent neutralizing capabilities *in vitro* and the previous studies showing their protective effects following SUDV infection in rhesus macaques, we sought to determine whether delayed treatment with a cocktail consisting of FVM04 and CA45 could protect cynomolgus macaques from developing severe disease following infection with SUDV. In this study, each animal was infected with 1,000 PFU of SUDV-Boniface and treated with intravenous injections of monoclonal cocktail (20 mg/kg/mAb) initiated on either day 4 with a second dose given on day 7 (early treatment group) or initiated on day 5 with a second dose given on day 8 (delayed treatment group). This schedule extends the window for effective treatment to 5 days after infection in comparison to previous studies showing the reversion of EVD in infected macaques following treatment with monoclonal antibodies and is similar to the previous study employing FVM04 and CA45 starting at day 4 (19).

**Fig 2:**
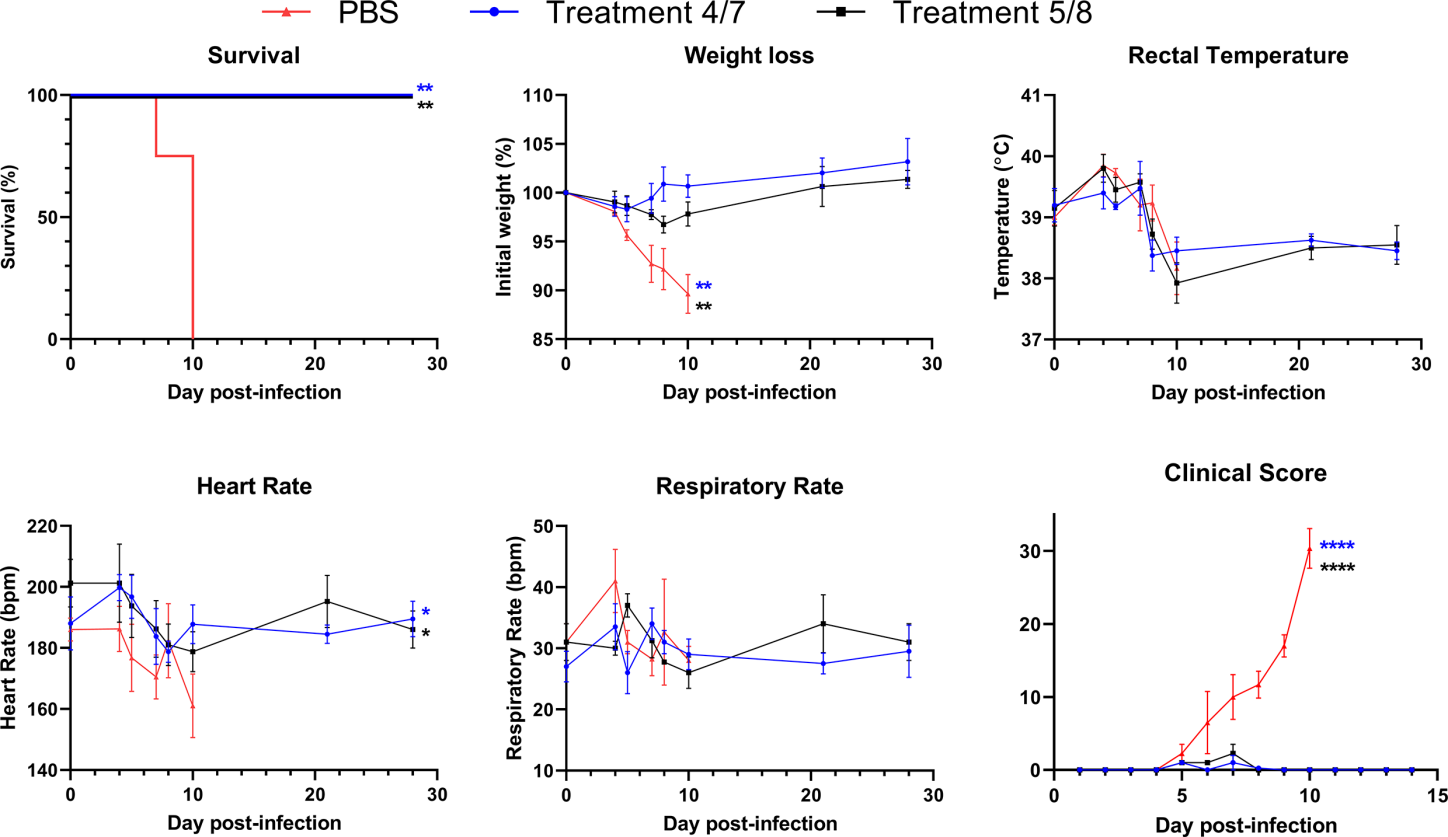
Survival, clinical score, and vital signs following SUDV infection. Cynomolgus macaques were infected with SUDV and then treated with a cocktail of monoclonal antibodies FVM04 and CA45 on either days 4 and 7 (blue) or days 5 and 8 (black). Survival, clinical score, and the indicated vital measurements are shown throughout the course of infection. *N* = 4 animals per group. Lines represent mean + SEM of each group. For survival, significance was assessed by log-rank test. For all other parameters, significance was assessed by one-way ANOVA with Dunnett’s post-test. *P < 0.05, **P < 0.01, ****P < 0.0001. The color of asterisk indicates significance for either the early treatment group (blue) or delayed treatment group (black) compared with the PBS group.

Clinical signs were monitored daily following infection and vital signs were recorded for each animal on specific days (Fig. 2). All treated animals survived the infection, while all four PBS-treatment animals had to be humanely euthanized after meeting euthanasia criteria based on clinical score, one on day 7, and three on day 10 (Fig. 2). The four PBS-treatment animals saw a steady increase in clinical score and significant weight loss beginning on day 5. Animals given treatment on days 5 and 8 showed some minor clinical signs and had mild weight loss, but recovered. All animals had increased body temperatures in the days following infection, regardless of treatment group or outcome, consistent with mild fever developing during acute infection. No differences were seen in the respiratory rate of infected animals throughout infection; however, the PBS-treatment group did show reduced heart rates, particularly on day 10, when the three remaining animals reached their terminal endpoint. Overall, both treatment regimens resulted in 100% survival and protection from severe disease.

**Fig 3:**
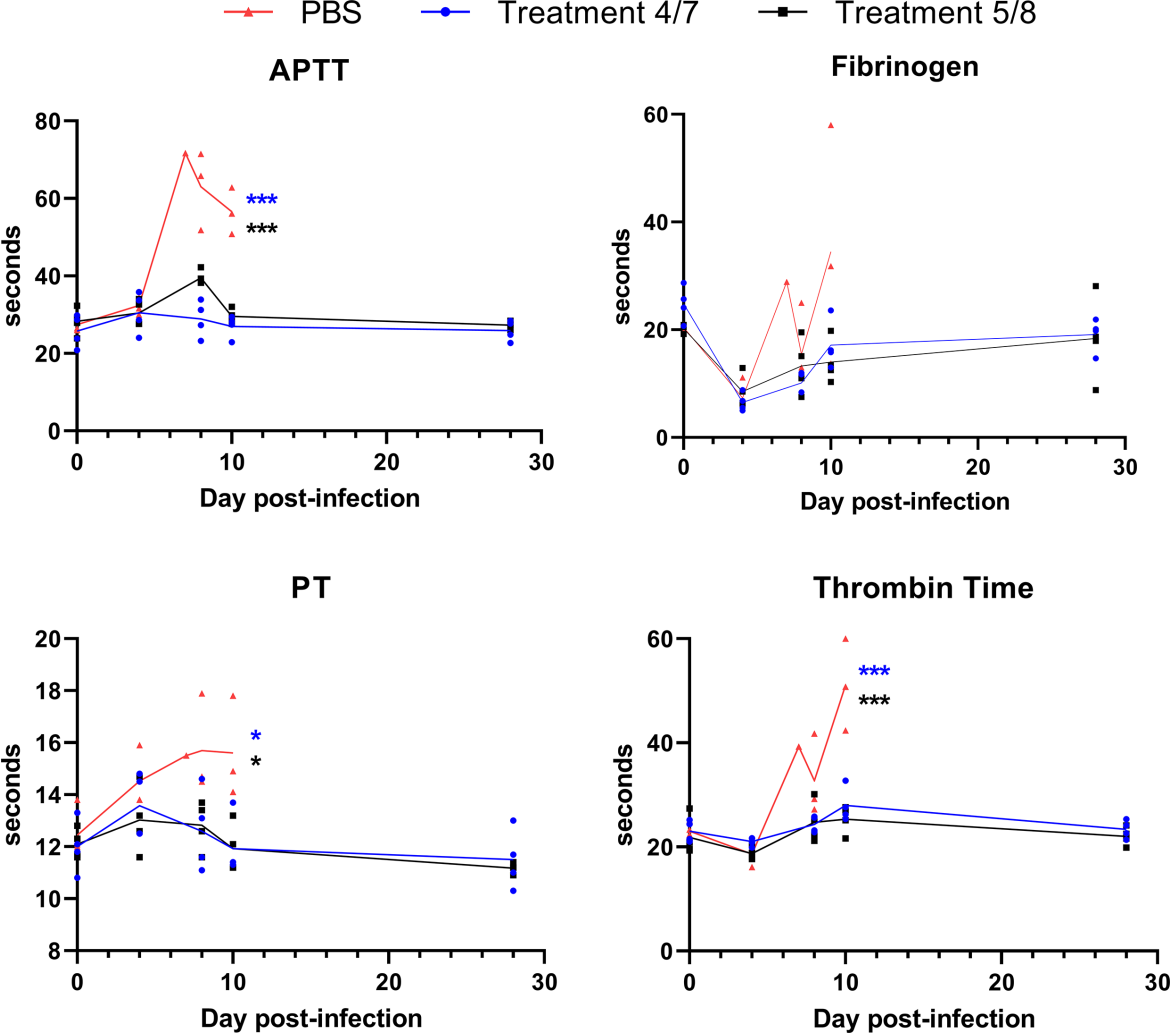
Coagulation parameters following SUDV infection. Blood coagulation time was assessed in cynomolgus macaques following SUDV infection in all groups. Blood plasma was collected on days 0, 4, 8, 10, and 28 post-infection. Values on day 7 are from only the one animal that was euthanized on that day. Activated partial thromboplastin time (aptt), fibrinogen, prothrombin time (pt), and thrombin time were measured. *N* = 4 per group, except PBS group on day 10 (*n* = 3). Lines represent mean values for each group, with individual values shown by symbols. Significance was assessed by one-way ANOVA with Dunnett’s post-test. *P < 0.05, ***P < 0.001. The color of asterisk indicates significance for either the early treatment group (blue) or delayed treatment group (black) compared with the PBS group.

One of the hallmarks of filovirus disease is the development of abnormalities in blood coagulation, leading to systemic hemorrhage (20). This is typified by increased clotting times and thrombocytopenia. We collected blood plasma from each animal prior to infection (Day 0 post-infection) and throughout infection and examined coagulation times as shown in Fig. 3. Activated partial thromboplastin time (APTT), prothrombin time (PT), and thrombin time were all significantly elevated in PBS-treated animals compared with the two treatment groups. Conversely, fibrinogen levels were not significantly higher in PBS-treatment animals compared with those receiving monoclonal cocktail treatment (Fig. 3). The lone PBS-treatment animal that reached its endpoint on day 7 had elevated fibrinogen, while two of the three remaining PBS-treated animals had elevated fibrinogen at day 10, their terminal time point. A similar trend was seen with thrombin time, which was elevated for that single animal on day 7, before increasing significantly on day 10. All four parameters remained relatively stable for the two treatment groups, aside from a small increase in APTT on day 8 in the delayed treatment group. These data suggest that SUDV infection leads to disrupted coagulation capabilities in infected macaques, which contributes to disease, and that monoclonal cocktail treatment can prevent this disruption.

**Fig 4:**
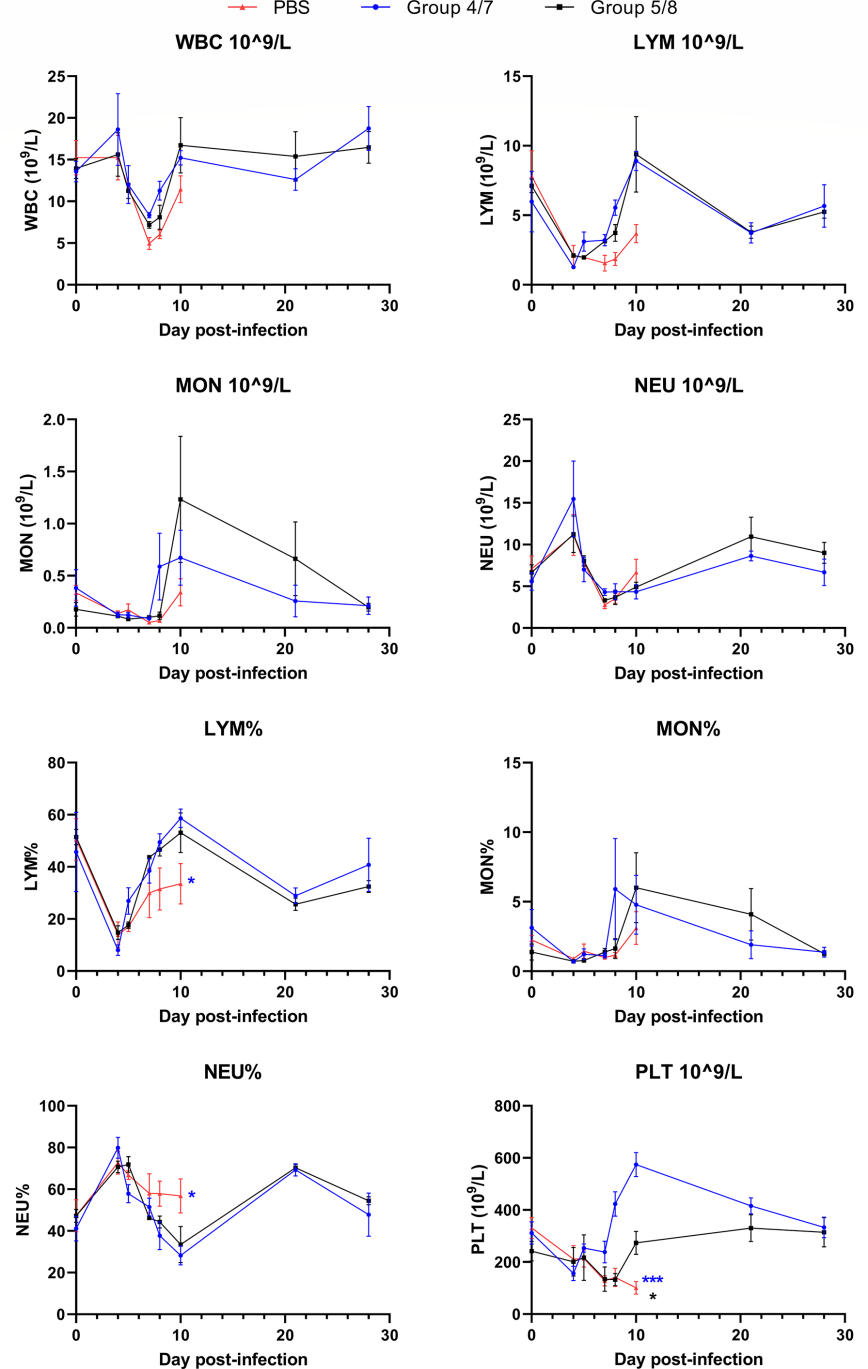
Hematological parameters following SUDV infection. Complete blood counts were assessed in cynomolgus macaques following SUDV infection in all groups. Whole blood was collected on days 0, 4, 5, 7, 8, 10, 21, and 28 post-infection. WBC, white blood cells; LYM, lymphocytes; MON, monocytes; NEU, neutrophils; PLT, platelets. *N* = 4 per group, except PBS group on days 8 and 10 (*n* = 3). Lines represent mean + SEM of each group. Significance was assessed by one-way ANOVA with Dunnett’s post-test. *P < 0.05, ***P < 0.001. The color of asterisk indicates significance for either the early treatment group (blue) or delayed treatment group (black) compared with the PBS group.

Filoviruses infect cells of the immune system, primarily those of the myeloid lineage, which can lead to reductions in various cell populations in circulation and the tissues due to migration and direct cell death (21–23). Lymphopenia is a common characteristic of filovirus disease and can be an indicator of poor outcomes following infection. We examined the number and percentage of certain cell types in the blood following infection, depicted in Fig. 4. Day 0 post-infection time point was taken prior to infection and represents baseline levels for each animal. Both treated groups showed similar trends throughout infection, having similar numbers of white blood cells, lymphocytes, monocytes, and neutrophils at all examined time points, with the only difference between these two groups being relative thrombocytopenia in the delayed treatment group which saw a rebound on day 10 (Fig. 4). Indeed, the lymphocyte, monocyte, and neutrophil % for these two groups also were highly similar throughout infection. In the PBS-treatment group, where all four animals succumbed to disease, a slight reduction in WBC and monocytes was seen on day 10, the terminal time point for three animals along with characteristic lymphopenia noticeable by day 7/8, though the difference in absolute lymphocyte counts was not statistically significant. However, lymphocyte % was significantly reduced in the PBS-treated group by day 10. While absolute numbers of neutrophils were not increased in this group, neutrophil % was higher in this group on the last two sampling days compared with both treatment groups. The PBS-treatment animals also experienced thrombocytopenia with notably reduced platelet count beginning on day 8. Our data suggest that following SUDV infection, all groups showed similar trends in several blood cell populations early, but following the initiation of treatment, treated groups were able to return to baseline levels and avoid deleterious changes that may impact infection outcome.

**Fig 5:**
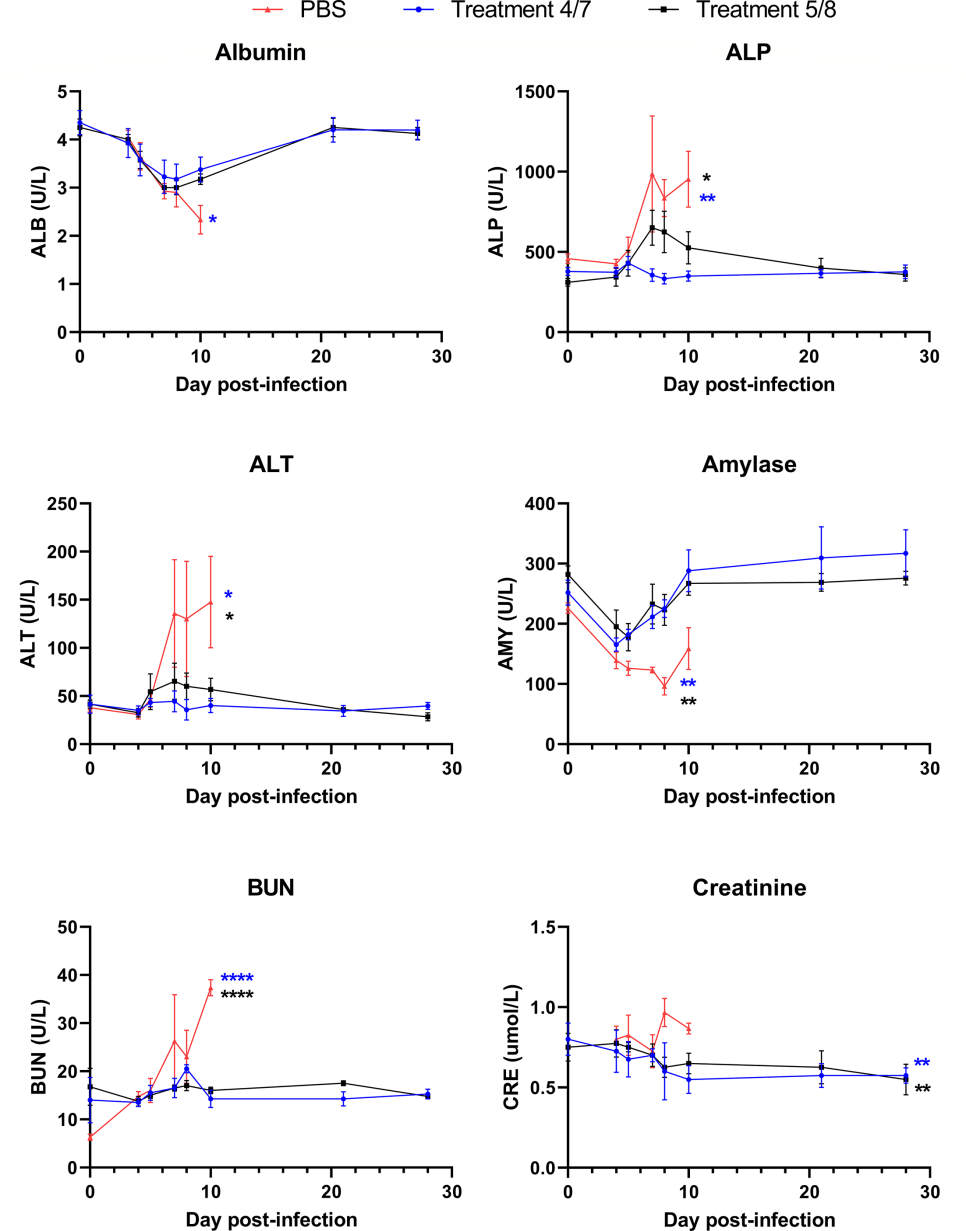
Serum biochemistry following SUDV infection. Analysis of serum biochemistry was assessed in cynomolgus macaques following SUDV infection in all groups. Serum was collected on days 0, 4, 5, 7, 8, 10, 21, and 28 post-infection. ALP, alkaline phosphatase; ALT, alanine aminotransferase; BUN, blood urea nitrogen. *N* = 4 per group, except PBS group on days 8 and 10 (*n* = 3). Lines represent mean + SEM of each group. Significance was assessed by one-way ANOVA with Dunnett’s post-test. *P < 0.05, **P < 0.01, ****P < 0.0001. The color of asterisk indicates significance for either the early treatment group (blue) or delayed treatment group (black) compared with the PBS group.

In addition to hematological perturbations, alterations in biochemical markers in the serum can be indicative of a disease state, including organ dysfunction or damage. At the same time points as above, we collected serum and examined several markers that might indicate this type of organ damage or dysfunction. As seen in Fig. 5, at the terminal endpoint, PBS-treatment groups had significantly increased alkaline phosphatase (ALP), alanine aminotransferase (ALT), blood urea nitrogen (BUN), and creatinine along with reduced serum albumin and amylase compared with both treated groups. The delayed treatment group showed an increase in ALP which was trending toward normal levels by day 10 and returned to baseline by day 21, after the acute phase of infection. Elevated ALP and ALT in PBS-treatment animals indicate liver damage that was spared in the early treatment group though present to some degree in the delayed treatment group. Low albumin at the terminal stage of disease in PBS-treatment animals is consistent with a lack of appetite during acute illness. Hypoamylasemia along with increased BUN and creatinine in PBS-treatment animals indicate pancreas and kidney dysfunction that is consistent with systemic filovirus disease. Overall, our data show that SUDV infection resulted in classical diagnostic markers indicative of severe filovirus disease in PBS-treatment animals, which was mostly averted in both treated groups, even when the administration of treatment was delayed.

**Fig 6:**
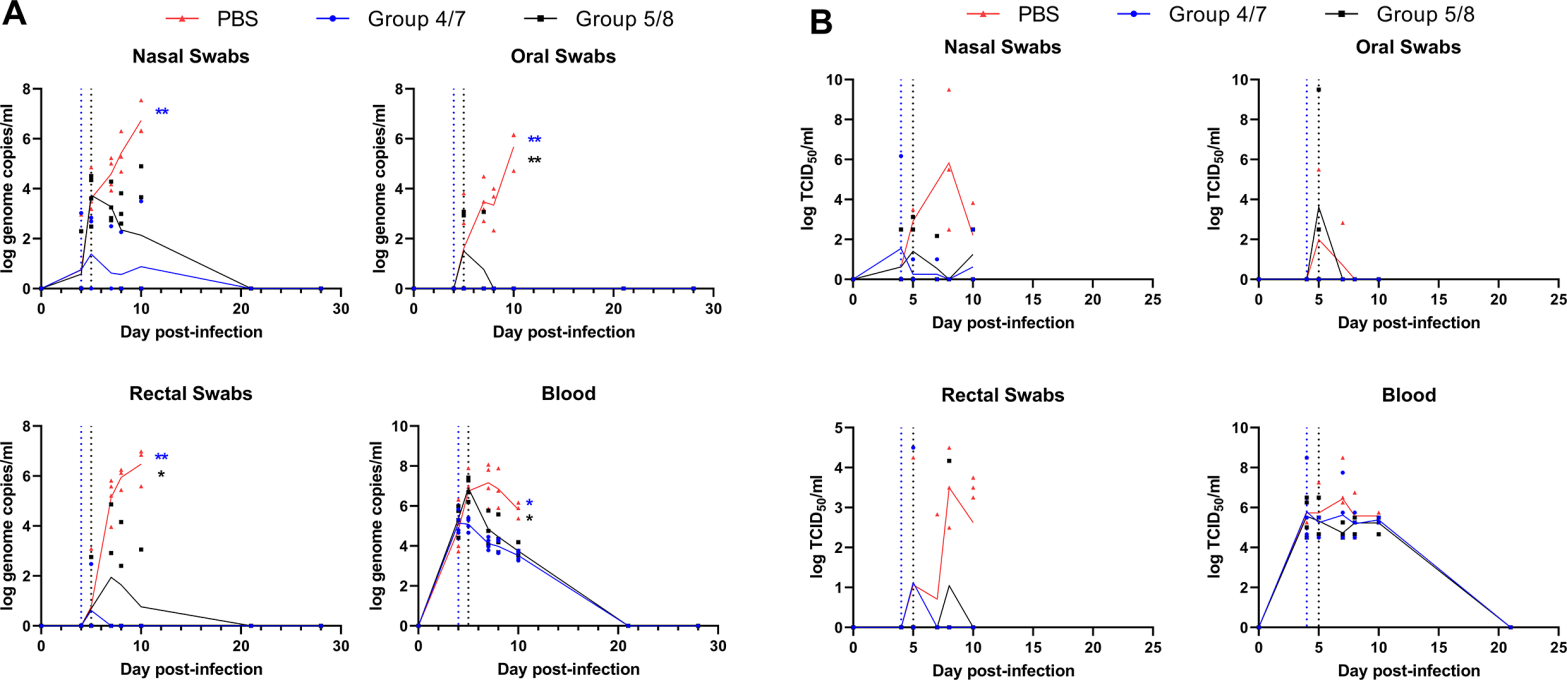
Viral loads during SUDV infection. Following SUDV infection, whole blood and oral, nasal, and rectal swabs were collected on days 4, 5, 7, 8, 10, 21, and 28 post-infection. Viral loads were assessed from samples collected on each day. (**A**) SUDV viral RNA quantified by RT-qPCR. (**B**) Infectious viral load quantified by TCID_50_. *N* = 4 per group, except PBS group on days 8 and 10 (*n* = 3). Lines represent mean values for each group, with individual values shown by symbols. Vertical dashed lines indicate the start of treatment. Significance was assessed by one-way ANOVA with Dunnett’s post-test. *P < 0.05, **P < 0.01. The color of asterisk indicates significance for either the early treatment group (blue) or delayed treatment group (black) compared with the PBS group.

In addition to focusing on the disease state, we collected serial blood samples as well as oral, nasal, and rectal swabs, to examine whether treatment affects the kinetics of viral replication in the blood and viral shedding in infected animals. Viral RNA was detectable in nasal swabs from most infected animals, including in both treatment groups where RNA increased following infection until treatment began, after which levels began to drop in treated animals, while RNA levels increased in PBS-treatment animals until they reached the study endpoint (Fig. 6A). RNA levels in nasal swabs were significantly higher in the PBS-treated group compared with the early treatment group, but not compared to the delayed treatment group, suggesting earlier treatment may help reduce viral shedding. Viral RNA in oral swabs was only detectable in the PBS treated and delayed treatment groups, with RNA first detectable on day 5 post-infection. Mean RNA levels increased steadily in PBS-treatment animals until they reached their terminal endpoint and were reduced significantly in the treated animals and undetectable by day 8 (Fig. 6A). Rectal swab RNA levels were also significantly higher in PBS-treated animals compared with both treated groups and followed similar kinetics, with increasing levels of RNA detectable until euthanasia in PBS-treatment animals, and low levels detectable in both treated groups, including low levels out until day 10 in the delayed treatment group. RNAemia was similar in all three groups up until the initiation of treatment, where RNA levels dropped sooner in the early treatment group compared to the delayed treatment group which continued to peak at day 5 and then decreased once treatment was initiated (Fig. 6A). In PBS-treatment animals, RNA levels in the blood remained high, with a peak seen at day 7, before beginning to drop before euthanasia. RNA levels were significantly higher in the PBS-treated group than both treatment groups, while RNA levels in the delayed treatment group trended higher than in the early treatment animals (Fig. 6). We also examined the presence of infectious viral loads in swabs, blood, and tissues of euthanized animals, where overall, infectious virus was not measured as consistently as viral RNA. Infectious viral load in swabs and blood followed a similar trend in each group to the RNA levels seen, peaking between days 5 and 10 before dropping in animals that had detectable virus (Fig. 6B). Only four animals had detectable virus in oral swabs during the course of the experiment. Titers in nasal and rectal swabs showed some differences between the PBS-treatment and cocktail-treated groups, but these were not statistically significant. Each treated group had a single positive rectal swab sample (one individual on day 5 in the early treatment group, one on day 8 in the delayed treatment group). Nasal swabs showed infectious virus more reliably than other swab samples; however, only a single early treatment animal had infectious titers in nasal swabs, and three delayed treatment animals had low levels seen early, but only a single positive nasal swab after the start of treatment (Fig. 6B). Infectious titers in the blood were consistently positive, and mean titers did not differ between the three groups, suggesting that the initiation of treatment did not immediately lower viremia that had been established after infection. Infectious SUDV was detectable in all tissues of PBS-treated animals that were examined following necropsy, including reproductive organs (Fig. S1).

While histopathological analysis of tissues was not performed due to all treated animals surviving infection, gross pathology was examined in tissues taken during necropsy after the PBS-treatment animals reached the humane endpoint. Representative lung tissue taken from a PBS-treatment animal showed multifocal to coalescing patchy reddened areas that involve all lung lobes. The lungs appear wet/shiny and fail to collapse with a likely combination of congestion, edema and hemorrhage (Fig. S2). In addition, all animals were monitored daily throughout infection, and in addition to the recording of vital signs and weight, clinical reports were completed each day outlining the condition of the animals (Fig. 2). Toward the late stages of disease, PBS-treatment animals developed significant petechiae characteristic of hemorrhage seen in filovirus infection. This was significant in the face, arms, legs, and groin (not shown). Monoclonal antibody cocktail-treated animals did not show any visible signs of disease such as petechiae and all had a clinical score at or below 1, except for a single animal in the early-treatment group with a score of 4 and a single animal in the delayed treatment group with a score of 6, both on day 7 (Fig. 2). Clinical scores for these animals were back to 1 and 0, respectively, by day 8.

**Fig 7:**
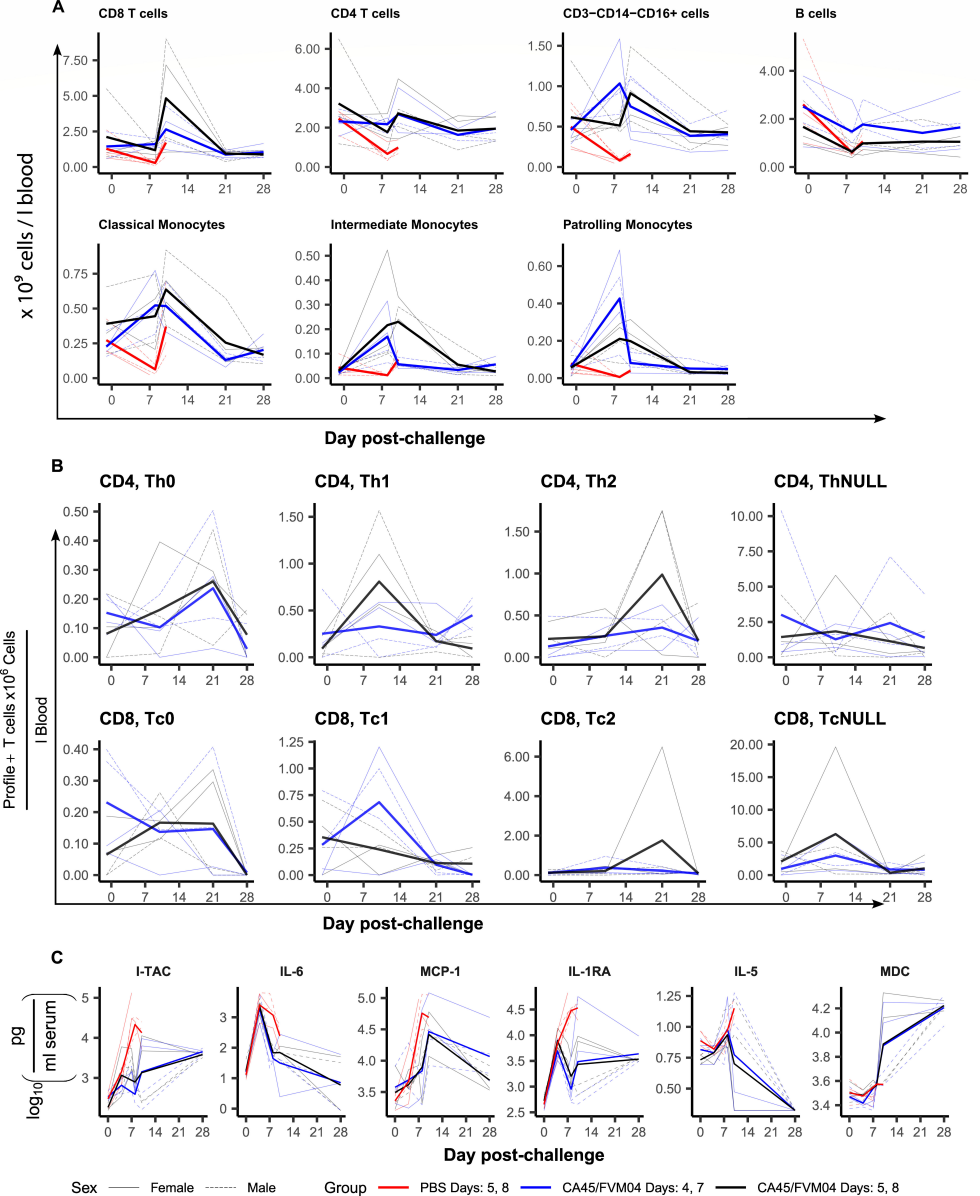
Immunological parameters following SUDV infection. (**A**) Measurement of blood lymphocyte populations throughout the study. (**B**) Measurement of T cell responses during the study, note the concentrations are expressed in million per liter. (**C**) Cytokines measured by Luminex assay. In every panel, thin lines represent individual animals while the thicker lines are group averages.

Whole blood was collected throughout the study and analyzed by flow cytometry to estimate the concentration of lymphocyte subpopulations in the blood (Fig. 7A). Overall, the groups treated with the antibody cocktail did not show the same decrease in various lymphocyte populations as measured in the PBS-treatment group. Additionally, a few differences between the two treatment groups were noted. CD8 T cells in the delayed treatment group peaked at almost twice the concentration of the early group, while CD4 T cells followed similar trajectories, on average. NK-like cells (CD3− CD14− CD16+ cells) peaked on day 10, except in females in the early treatment group, with similar levels in both treated groups. Both treatment groups had a slight decrease in B cells at early times, but the difference between them stayed relatively constant throughout the study. Classical monocytes (CD14++, CD16−) increased earlier in early-treatment females than in other treated animals with both groups returning below baseline by day 21. The intermediate monocytes (CD14+, CD16+) increased early in the early-treatment females but did not increase much in early-treatment males while late-treated animals showed more variation with one female reaching a high peak on day 8, one male and one female having peaks on day 10, and one male with little change in intermediate monocytes. The patterns of patrolling monocytes (CD14+, CD16++) had more consistent patterns with three of four early-treatment animals reaching high peaks by day 8, which returned to baseline by day 10 and 3 of the four delayed treatment animals having lower but more sustained responses.

The Th polarization of the CD4 and CD8 T cells was assessed by stimulating 100 µL of whole blood overnight and then adding golgi inhibitors for 6 h. The cells were stained for cytokines and analyzed by flow cytometry (Fig. 7B). The Th0 (CD4) and Tc0 (CD8) profiles of T cells secreting both interferon gamma (IFNγ) and IL-4 remained relatively low throughout the experiment but some animals did see increases on days 10 or 21. In CD4 T cells, the delayed treatment animals showed an interesting switch between Th1- (IFNγ+, IL-4−) and Th2-dominant (IFNγ−, IL-4+) profiles between days 10 and 21. Meanwhile, the early treatment animals had a weaker response in both profiles. In CD8 T cells, 2 of the early treatment animals displayed a strong Tc1 (T1 CD8 T cells) response while the other animals did not change much from baseline or decreased. One female of the delayed treatment group had a very strong Tc2 (Type 2 CD8 T cells) response on day 21 and 1 early-treatment male had an elevated CD8 Tc2 response on day 10. The ThNULL (and TcNULL; IFNγ−, IL-4−, CD69/TNF/CD107a response) response was highly variable in CD4 T cells but peaked on day 10 for the CD8 T cells.

Serum cytokine levels were measured by Luminex. All 28 cytokines measured increase by day 10, with some having earlier peaks (Fig. 7C; Fig. S3). Six cytokines showed interesting patterns (Fig. 7C). Interferon-inducible T-cell alpha chemoattractant (I-TAC), IL-6, and monocyte chemoattractant protein 1 (MCP-1) are pro-inflammatory cytokines and chemokines where the PBS-treatment animals showed an earlier and/or sustained increase compared to the treated animals. The PBS-treatment animals also had higher levels of the anti-inflammatory cytokine IL-1RA, which increased until they reached the humane endpoint. An interesting pattern appeared in the treated animals and was constant between the two groups, consisting of a sex-difference in cytokine levels on day 10 which disappears by day 28. This is most readily observable in I-TAC, IL-1RA, IL-5, and macrophage-derived cytokine (MDC), but also present to a lesser extent in EGF, FGF-basic, and RANTES (Fig. S3).

## DISCUSSION

With the continued emergence and re-emergence of filoviruses causing outbreaks across sub-Saharan Africa, there remains a need for efficacious and available therapeutics in case of a large outbreak or continued spread of infections. While there is currently an FDA-approved vaccine and 2 FDA-approved monoclonal antibody products for EBOV that has been shown to protect against EVD, options for other ebolavirus species or filoviruses are lacking (24). During the West African EBOV outbreak, the use of ZMAPP, Inmazeb (REGN-EB3), and Ebanga (ansuvimab-zykl) to treat EBOV-infected patients highlighted the advancement that monoclonal antibody therapies have made. Further development of antibody therapeutics has provided more effective options for EBOV that have reached approval for clinical use (17–20). However, faced with additional ebolavirus strains that may cause outbreaks warrants the development of additional strain-specific or, more optimally, broad-spectrum therapeutics targeting multiple ebolavirus species. Specifically, antibodies or antibody cocktails that show pan-ebolavirus or pan-filovirus reactivity are of particular interest, as their development could provide an option for treatment regardless of the infecting ebolavirus species. Previously, the NHP-derived chimeric antibodies FVM04 and CA45 have shown pan-ebolavirus protective capabilities in multiple species, including complete protection against lethal disease caused by EBOV, SUDV, and Bundibugyo (15–19). Here, we have expanded upon previous findings by showing that delayed treatment with a cocktail consisting of these two antibodies completely protects cynomolgus macaques from lethal disease in a fully lethal model of SUDV infection. Cynomolgus macaques were chosen because they provide a stringent, reliable model that closely mimics the human disease course following infection with Filoviruses (25–28). They have been used extensively to model filovirus disease and have been critical in evaluating medical countermeasures including vaccines and specific therapeutics. This reliable recapitulation of human disease allowed for us to test our cocktails within realistic treatment windows, as has been done previous for EBOV infections in NHPs (29).

Our *in vitro* neutralization data is consistent with previous reports for both CA45 and FVM04 against SUDV (15, 17, 18). Our calculated IC_50_ value of 90 ng/mL for CA45 against SUDV Boniface is similar to the previously reported EC_50_ of 78 ng/mL against authentic SUDV, while our FVM04 IC_50_ of 1.29 is similar to previous reports using both recombinant vesicular stomatitis virus expressing SUDV GP and authentic SUDV (15, 17). We also tested each antibody against SUDV-Gulu, which showed a slight resistance to neutralization by FVM04 compared with SUDV-Boniface (Fig. 1). Interestingly, the IC_50_ for CA45 against SUDV-Gulu was nearly 10-fold greater than for SUDV-Boniface. This may be due to differences between SUDV-Boniface and SUDV-Gulu at key binding residues for CA45 within the fusion loop of the GP.

A previous study showed full protection from lethality in SUDV-infected rhesus macaques given FVM04/CA45, however, that study included only two PBS treated animals, one of which survived the infection (19). In this study, all treated animals survived SUDV infection, with little to no morbidity noted throughout the experiment. In contrast, all PBS-treated groups rapidly deteriorated after day 5 post-infection, with one animal requiring euthanasia on day 7, and the remaining three requiring euthanasia on day 10 (Fig. 2). Regardless of the onset of monoclonal cocktail treatment (day 4 or day 5), treated animals fared remarkably well, with only two animals, one in each treatment group, having a recorded clinical score greater than 1 on only 1 day after infection. Among treated animals, very few perturbations in the assessed parameters were noted. The delayed treatment group had minor weight loss but recovered to baseline weight by day 21. This group also showed a trend toward minor thrombocytopenia, similar to that seen in PBS-treated animals before seeing an increase in platelet count. They also showed an increase in ALP, indicating the potential onset of disease, before falling back to baseline levels. The early treatment group did not see alterations in most parameters examined. These differences are likely due to the onset of severe disease beginning around day 5 post-infection, where PBS-treatment animals began to show signs of illness and rapidly become ill. Overall, similar trends are seen in all groups up until days 4 or 5, at which point clinical parameters and signs of disease start to appear in PBS-treated animals that are not seen in the treated animals. Treatment onset on day four likely prevents further replication and spread of SUDV and the ensuing systemic response leading to morbidity that begins around this time in PBS-treated animals. Animals treated on day 5, with an extra day of untreated viral replication come closer to exhibiting clinical disease (clinical parameters that fall out of range with normal) before treatment. Inhibition of viral replication by treatment and the developing immune response likely act together to prevent severe illness from occurring. Given that the disease course for SUDV infection in NHPs is slightly longer than what is seen in EBOV infection, the onset of treatment in this study was at or just before halfway to the median time of death of 10 days. In EBOV infection, treatment beginning on day 5 can rescue NHPs from lethal disease that occurs on days 7–8; therefore, it would be relevant and worthwhile to determine the latest point at which FVM04/CA45 treatment can prevent lethal SUDV infection in cynomolgus macaques (29). Future testing of FVM04/CA45 treatment regimens beginning on day 6 post-infection and later would be useful to determine whether complete protection can be maintained. Delayed treatment until the point that animals have started to show signs of disease would be relevant for the treatment of symptomatic SUDV-infected patients.

Following SUDV infection, all groups showed similar levels of viral shedding detected by oral, nasal, and rectal swabs, up until the time of treatment, after which viral RNA levels dropped significantly in treated groups while they continued to increase until termination in PBS-treatment animals (Fig. 6). Expectedly, intravenous antibody treatment resulted in reductions in detectable viral RNA in the blood; however, we did not see a reduction in infectious virus in the blood following treatment onset (Fig. 6). The delayed-treatment group had slightly higher levels of viral RNA present in the blood compared with the early treatment animals, though levels of infectious virus were similar, suggesting that delaying treatment by one day did not lead to significantly more viral replication. However, treatments also appeared to reduce viral shedding, indicating that either infused antibody can migrate to mucosal surfaces and neutralize viral infection, or systemic treatment prevents viral replication and systemic spread by preventing virus from making it to the mucosa to be shed. These results have important implications for viral transmission; however, the presence of viral RNA or infectious virus was not examined in other bodily fluids that may be important for human-to-human transmission. Because we did not have treated animals for euthanasia at the same time points that PBS-treated animals were euthanized, we cannot compare tissue viral titers, however all PBS-treatment animals had high viral titers in all tissues examined, indicating systemic spread of the virus. This was also supported by changes in the clinical and biochemical parameters indicative of the systemic disease. Another caveat to this study was the lack of a non-specific isotype-matched antibody cocktail to use as a control treatment group. Although we used PBS as a control treatment group for comparison to the antibody-treated animals, our CA45 and FVM04 neutralization data along with the observed reduction in SUDV titers and morbidity in only the SUDV-infected and antibody-treated animals supports that protection is likely due to the specificity of these antibodies towards ebolavirus GP. Inclusion of isotype-matched controls in future studies should be considered. Previous animal studies where FVM04 or CA45 were used as a treatment, including rodent studies, did not include serial tissue collections or matched FVM04 and/or CA45-treated and PBS-treated animals for comparison of tissue titers. Future studies examining the impact of this cocktail treatment on systemic replication of SUDV or other ebolaviruses would be worthwhile.

Here, we have provided further strong evidence of the clinical efficacy in cynomolgus macaques of FVM04/CA45 monoclonal antibody cocktail treatment against SUDV infection in a delayed treatment scenario. Our data adds another species in which treatment was fully protective against lethal disease, as well as protective against most clinical signs and morbidity. Given the unmet need for further development of therapeutic options for the treatment of ebolavirus and filovirus infections, our study indicates that it is important to continue the development of FVM04/CA45, and that these mAbs may provide an option for treatment against multiple ebolavirus species. Discovery and development of antibody cocktails, containing multiple distinct monoclonal antibodies that react against diverse parts of the GP and are able to work synergistically to neutralize virus similar to that seen with FVM04 and CA45, should be pursued and prioritized.

## MATERIALS AND METHODS

### Study design

Twelve cynomolgus macaques (*Macaca fascicularis;* equal male and female, distributed equally in three groups of four) were acquired through commercial suppliers and acclimated for at least 1 week upon arrival at the NML prior to the beginning of the study. All manipulations and sampling were performed under ketamine anesthesia (10 mg/kg) followed by maintenance under inhalation isoflurane. Pre-bleeds were collected from the femoral vein at the onset of the experiment, and for viral challenge all animals were injected intramuscularly with 1,000 plaque-forming units of SUDV-Boniface in the quadriceps muscle. Four animals received PBS as mock treatment on days 4 and 7 to serve as controls. For treatment with FVM04/CA45 monoclonal antibody cocktail, animals received an intravenous bolus of 20 mg/kg in normal saline, with the volume adjusted according to the individual weights of each animal. Total volumes instilled at each time point ranged from 4.5 to 5.3 mL. One group received treatments on days 4 and 7, and another received treatments on days 5 and 8.

Animals were provided water *ad libitum* and were monitored twice daily for signs of illness or distress. Clinical scoring was performed based on food and water intake, posture and activity, weight loss, temperature, respiration rate, presence of visible rash, or hemorrhage, using a predetermined scoring chart. Animals were fed daily a diet consisting of primate chow (LabDiet) as well as fresh fruits and vegetables. All animals were sampled on days 4, 5, 7, 8, 10, 21, and 28. Sampling included femoral vein bleeds for whole blood, serum, and plasma (days 4, 8, 10, and 28 only. Day 7 upon euthanasia for one animal), as well as oral, nasal, and rectal swabs. For swab collection, swabs were placed in DMEM containing 1% fetal growth serum with 1% penicillin-streptomycin and 1% L-glutamine, and then swabs were performed and placed back into the media. At all sampling time points, vital signs (heart rate, respiratory rate, rectal temperature) and weight loss were recorded. For euthanasia, when animals in the PBS-treatment group reached their clinical endpoint animals were sampled as previous and blood was collected via cardiac puncture followed by intracardiac injection of pentobarbital (100 mg/kg). All animals in the treated groups were sampled on day 28 and kept for future studies.

### Cells and viruses

CV-1 cells (ATCC) were cultured in Minimal Essential Medium (MEM) (Hyclone) supplemented with 10% Fetal Bovine Serum and Vero E6 cells were cultured in Dulbecco’s Modified Eagle’s Medium supplemented with 5% Bovine Growth Serum Supplemented Calf (Hyclone). Cells were cultured at 37°C with 5% CO2. A passage 3 stock of wild-type Sudan virus, isolate Boniface (GenBank accession no. FJ968794.1) was prepared in Vero E6 cells. The virus titer was determined by TCID_50_ assay on Vero E6 cells. Rocky Mountain Laboratories kindly provided Sudan virus, strain Gulu.

### *In vitro* neutralization of SUDV

Antibodies FVM04 and CA45 were produced as previously described (18). Two-fold serial dilutions of FVM04 or CA45 were incubated for 1 h with 1,000 TCID_50_/mL of the Boniface or Gulu strains of Sudan virus at 37°C. One hundred microliters of antibody-virus mixtures was plated in triplicate on Vero E6 cells (SUDV_Gulu) or CV-1 cells (SUDV_Boniface). Cytopathic effect due to viral replication was read on day 14 post-infection. The analysis was carried out in R using the CmdStanR package. A Bayesian probit model was fit to the data, using weakly informative priors: normal (0, 5) on the slope and a normal distribution that allows all tested concentrations as the midpoint (IC_50_). Four chains were run for 5,000 sampling iterations, summary values were calculated from all samples. The curve corresponding to the median posterior value of each parameter as well as curves representing 100 random posterior samples were plotted.

### Blood coagulation, complete blood counts, and serum biochemistry

Complete blood counts (CBC) and hematological analysis were obtained using a VetScan HM5 (Abaxis Veterinary Diagnostics), as per manufacturer’s instructions. Serum biochemistry values were determined with a VetScan VS2 (Abaxis Veterinary Diagnostics) using complete diagnostic profile disks according to manufacturer’s instructions. Coagulation was assessed using a STart4 hemostasis analyzer (Diagnostica Stago) per manufacturer instructions.

### Quantification of SUDV by RT-qPCR and TCID_50_

For measurement of viral titers in swabs and tissues, TCID_50_ assays were performed. Samples were frozen at −80°C for storage and then were thawed. In the case of tissues, each was placed in MEM supplemented with 1× L-glutamine and 1% FBS and homogenized with 5 mm stainless steel beads in a Bead Ruptor Elite Tissue Homogenizer (Omni) for 1 min at a frequency of 4 m/s. Homogenates were clarified by centrifugation at 1,500 × *g* for 10 min and 10-fold serial dilutions of tissue homogenates were made in MEM supplemented with 1× L-glutamine, 1% FBS, and a 2× dose of penicillin/streptomycin. For swabs, each swab was performed and stored in the same medium as above. Swab media were clarified by centrifugation at 1,500 × *g* for 10 min and 10-fold dilutions were made in the same media as above. Whole blood samples were diluted in 10-fold serial dilutions using the same media as above. For the assay, all dilutions were added to 90%–100% confluent CV-1 cells in triplicate wells and cytopathic effect was read on day 10 post-infection. TCID_50_ values per mL or gram of tissue were calculated using a modified Reed and Muench method (30).

For quantification of viral RNA levels, RNA was extracted from whole blood or swab media using a viral RNA mini kit (Qiagen) as per manufacturer’s instructions. Detection of SUDV by RT-qPCR was performed on a QuantStudio 5 instrument (Applied Biosystems) using a TaqPath 1-step RT-qPCR Master Mix (Applied Biosystems) and primers/probes specific for the L gene of SUDV (Forward, CAGAAGACAATGCAGCCAGA; reverse, TTGAGGAATATCCCACAGGC; probe, 6-carboxyfluorescein [FAM]-CTGCTAGCT[ZEN]TGGCCAAAGTCACAAG-Iowa Black quencher; all from IDT). Oligonucleotide concentrations were 400 nM. Stages were as follows: UNG incubation (25°C for 2 min), reverse transcription (53°C for 10 min), polymerase activation (95°C for 2 min), followed by amplification (40 cycles of 95°C for 3 s and 60°C for 30 s).

### Immunophenotyping by flow cytometry

A detailed protocol is available via Protocols.io (31) (the viability staining was not performed for this experiment). Briefly, whole blood samples were incubated for 10 min at room temperature with Human TruStain FcX before staining with CD4 PerCP-Cy5.5 (clone L200, BD Biosciences), CD45RA PE-CF594 (clone 5H9, BD Biosciences), CD3 Alexa Fluor 700 (clone SP34-2, BD Biosciences), CCR7 BV711 (clone G043H7, BioLegend), CD8 BV786 (clone RPA-T8, BD Biosciences), CD45 Alexa 488 (clone D058-1283, BD Biosciences). Erythrocytes were removed via incubation with 1X BD Biosciences FACS Lysing solution before fixation and permeabilization of the remaining cells with BD Biosciences Cytofix/Cytoperm solution and removal from CL-4. Following removal from containment, all samples were interrogated using a BD Biosciences LSRII flow cytometer; fluorescence spillover was corrected using singly-stained compensation beads (Invitrogen UltraComp eBeads or ArC Amine Reactive Beads). Data were analyzed using FlowJo version 10 software. The counts for each population were converted to cell concentration by expressing them as a percentage of the CD45+cell population and assuming that population corresponds to the WBC parameter measured by the HM5. A gating strategy is presented in Fig. S4.

### T cell assay by flow cytometry

A detailed protocol is available via Protocols.io (32) (the viability staining was not performed for this experiment). Briefly, whole blood samples were incubated overnight in RPMI 10% FBS 50 µM β-mercaptoethanol with or without 5 µg/mL of formaldehyde-inactivated SUDV. Brefeldin A, monensin, and anti-CD107a BV421 (clone H4A3, BioLegend) were added for 6 h. The samples were incubated at room temperature for 10 min with Human TruStain FcX before staining with CD4 PerCP-Cy5.5 (clone L200, BD Biosciences), CD45RA PE-CF594 (clone 5H9, BD Biosciences), CD3 Alexa Fluor 700 (clone SP34-2, BD Biosciences), CCR7 BV711 (clone G043H7, BioLegend), CD8 BV786 (clone RPA-T8, BD Biosciences), CD69 BV605 (clone FN50, BioLegend). Erythrocytes were removed via incubation with 1X BD Biosciences FACS Lysing solution before fixation and permeabilization of the remaining cells with BD Biosciences Cytofix/Cytoperm solution and removal from CL-4. Following removal from containment, all samples stained with antibodies to IL-2 Alexa Fluor 488 (clone MQ1-17H12, BioLegend), IL-4 PE (clone 8D4-8, BioLegend), TNF PE-Cy7 (clone Mab11, BioLegend), and IFNγ APC (clone B27, BioLegend). Fluorescence spillover was corrected using singly-stained compensation beads (Invitrogen UltraComp eBeads or ArC Amine Reactive Beads). Data were analyzed using FlowJo version 10 software. The counts for each population were converted to cell concentration by expressing them as a percentage of the CD4 or CD8 T cell populations and assuming that population corresponds to the CD4 and CD8 T cell counts of the immunophenotyping. A gating strategy is presented in Fig. S5.

### Detection of serum cytokines by Luminex

Cytokine responses in serum were examined using the Cytokine Monkey Magnetic 29-Plex Panel for Luminex Platform (ThermoFisher Scientific). Cytokines that were analyzed included: G-CSF, IFN-γ, IL-10, IL-12, IL-17A, IL-2, IL-4, IL-6, IL-8, MCP-1, MIP-1α, MIP-1β, RANTES, TNF-α, EGF, Eotaxin, FGF-2, GM-CSF, HGF, IL-1β, IL-1RA, IL-15, IL-5, IP-10, I-TAC, MDC, MIF, MIG, VEGF-A. The procedure was as described by the manufacturer’s recommendations. Serum samples were diluted 1:8 and test plates were run using a Luminex MAGPIX instrument. Plots were produced using R.

### Statistical analysis

All results were graphed using either GraphPad Prism 9 software or R. Statisical significance was assessed by log-rank test for survival, or by one-way analysis of variance (ANOVA) with Dunnett’s post-test for multiple comparisons. Each treatment group was compared with the PBS control group. For parameters where there were statistically significant differences between groups, colored asterisks matching each group indicate significance levels.

## Data Availability

All data are available upon request, and inquiries should be sent to darwyn.kobasa@phac-aspc.gc.ca.

## SUPPLEMENTAL MATERIAL

Supplemental figures jvi.01242-23-s0001.pdf Fig. S1-S5.

ASM does not own the copyrights to Supplemental Material that may be linked to, or accessed through, an article. The authors have granted ASM a non-exclusive, world-wide license to publish the Supplemental Material files. Please contact the corresponding author directly for reuse.
