# Supplementary material for: Delayed treatment of cynomolgus macaques with a FVM04/CA45 monoclonal antibody cocktail provides complete protection against lethal Sudan virus infection
Source: J Virol. 2024 Jul 16;98(8):e01242-23. doi: 10.1128/jvi.01242-23 (PMC11334508; doi:10.1128/jvi.01242-23)
Supplement: Supplemental figures — Fig. S1-S5. [file jvi.01242-23-s0001.pdf]

**Supplementary Materials**

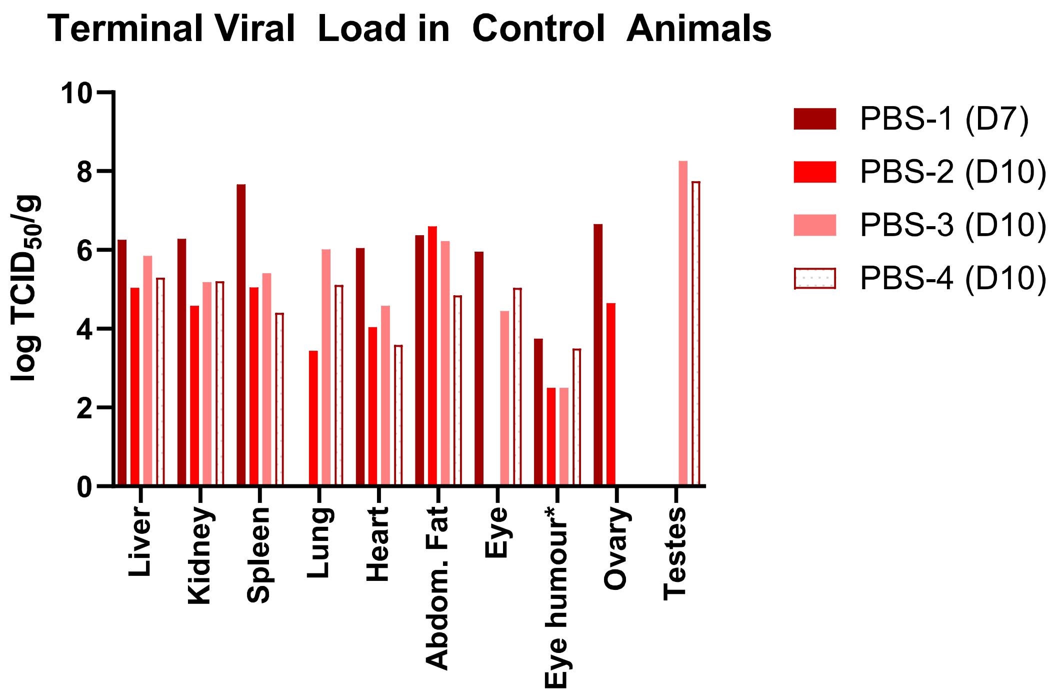


**Fig. S1. Tissue viral load at terminal end point in SUDV infected animals.** Infectious viral load in the tissues of each PBS treated animal at their terminal end point. Each bar represents an individual animal.

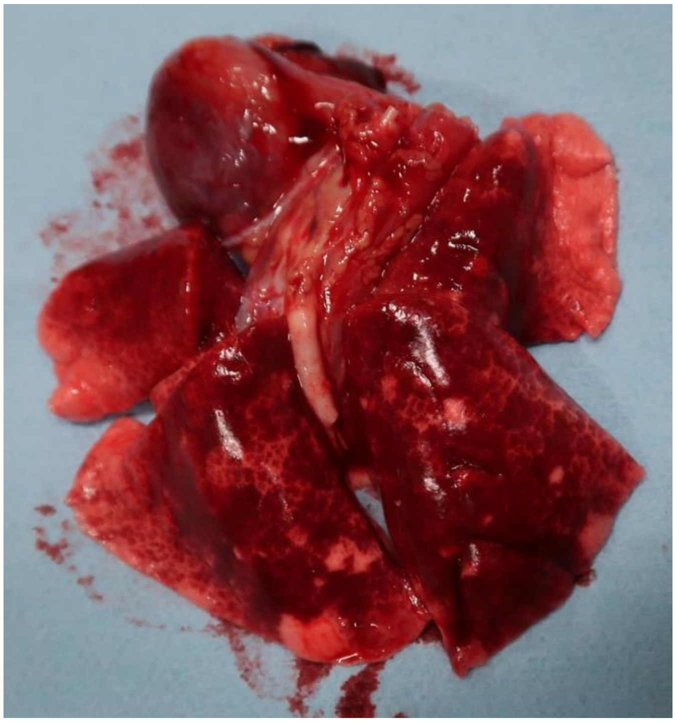

**Fig. S2. Representative gross pathology lung lesions in SUDV infected, PBS control animals.**

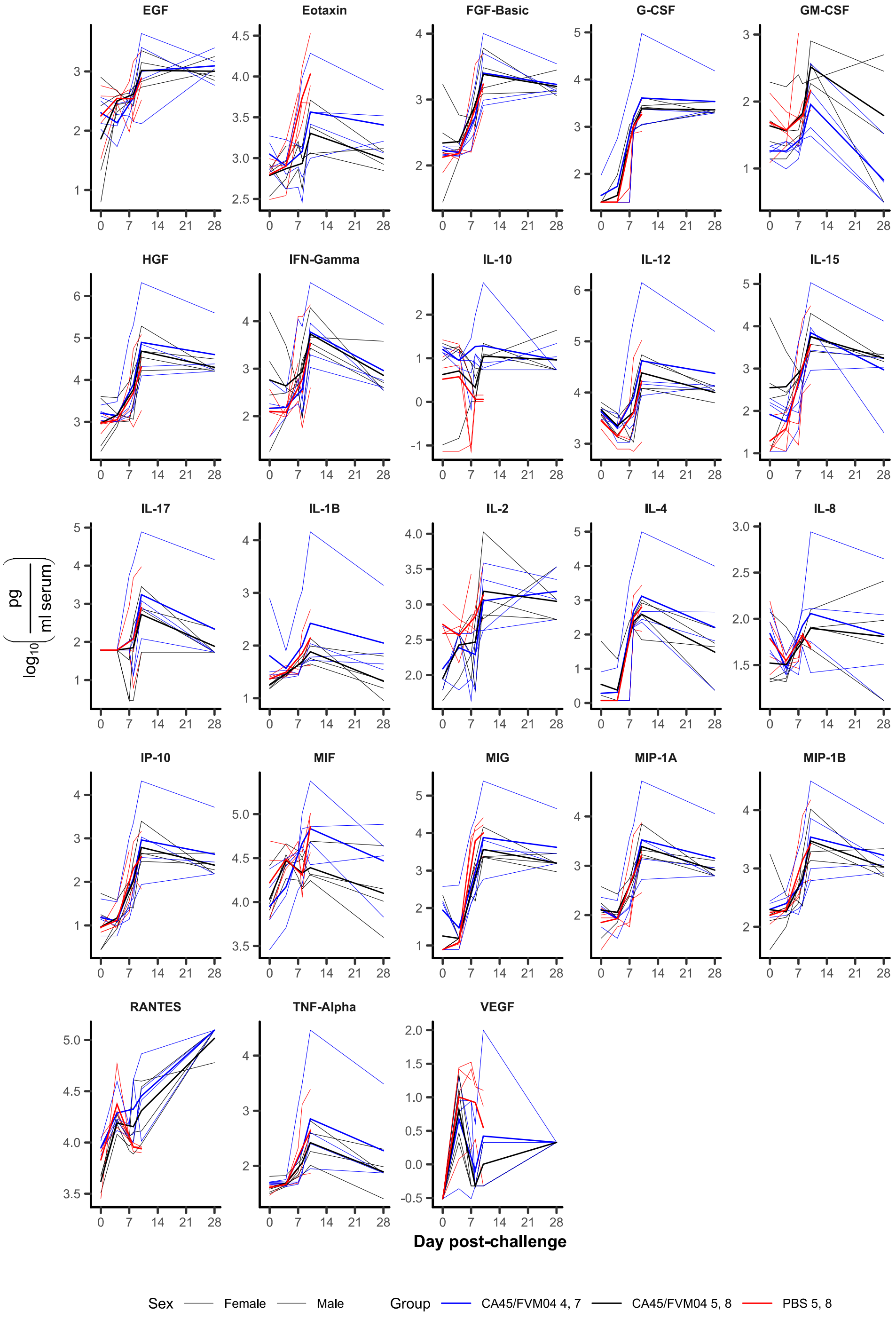


**Fig. S3. Additional cytokines.** Additional cytokines measured by Luminex assay. In every panel, thin lines represent individual animals while the thicker lines are group averages.

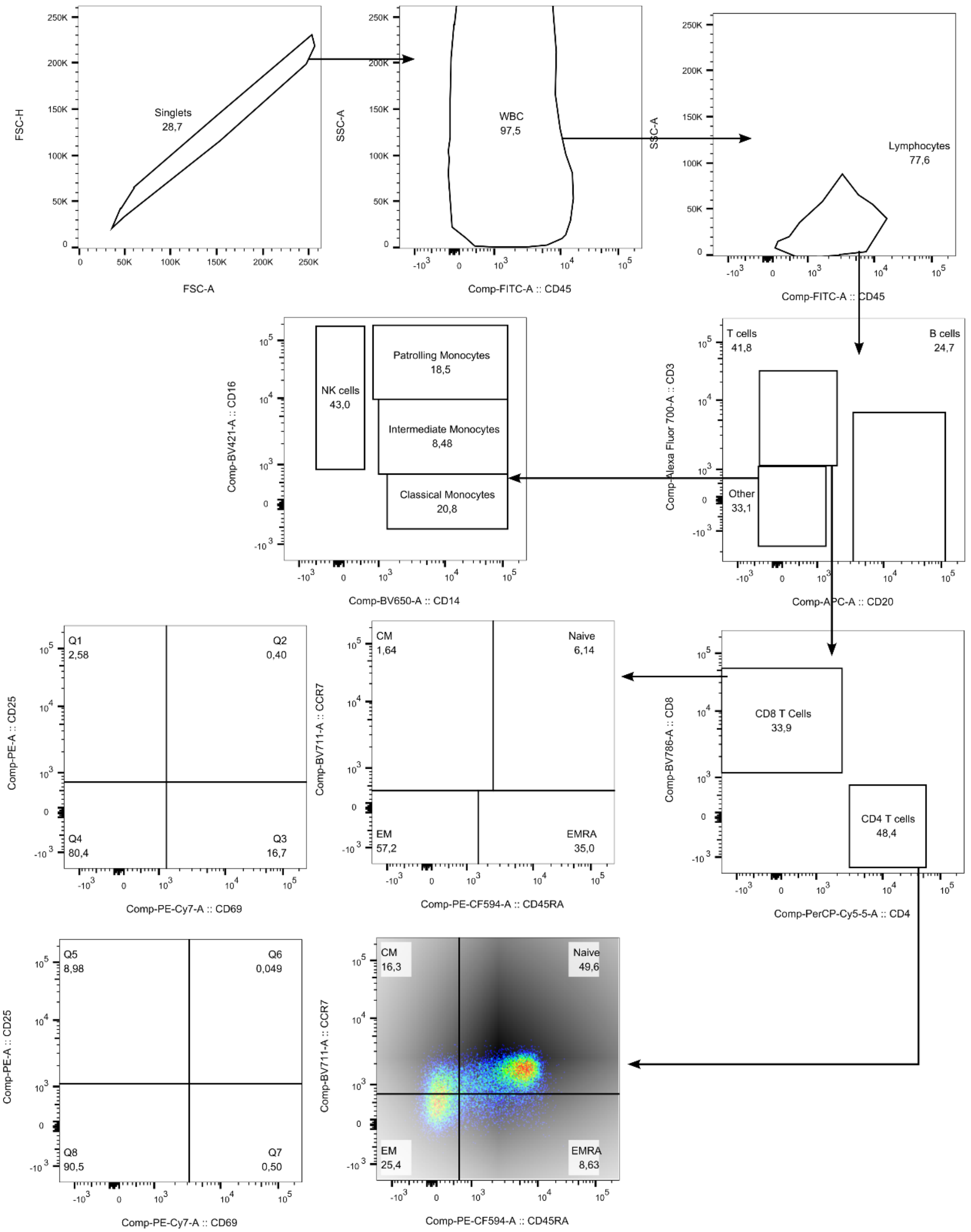


**Fig. S4. Flow cytometry gating strategy for immunophenotyping.**

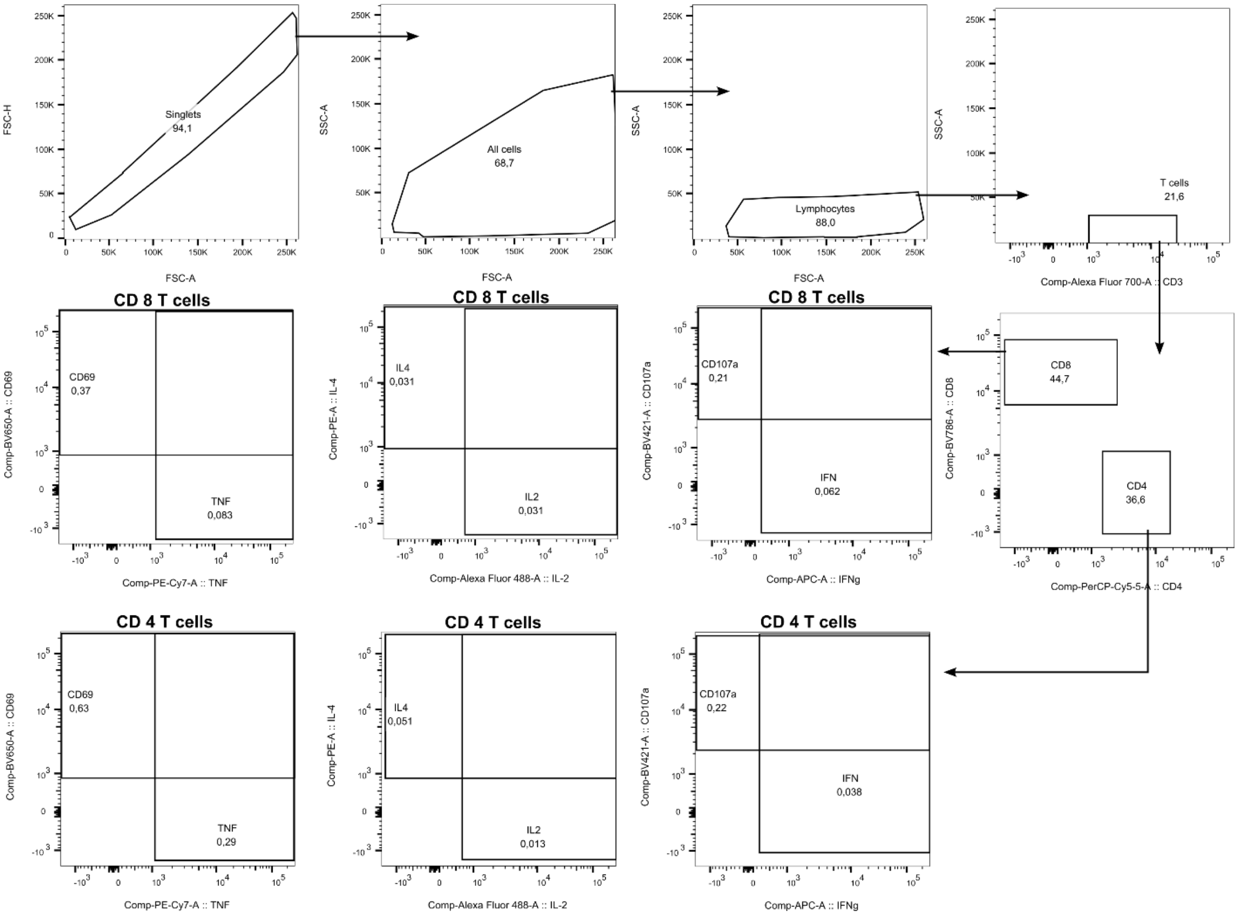


**Fig. S5. Flow cytometry gating strategy for T cell assay.**
